# LIFECOURSE DETERMINANTS OF DEPRESSION AMONG OLDER MEXICAN ADULTS

**DOI:** 10.1093/geroni/igac059.3041

**Published:** 2022-12-20

**Authors:** Paige Downer, John Prochaska, Rebeca Wong

**Affiliations:** University of Texas Medical Branch, League City, Texas, United States; University of Texas Medical Branch, Galeveston, Texas, United States; University of Texas Medical Branch, Galveston, Texas, United States

## Abstract

Older adults’ exposure to poverty, poor health, and negative life events over their lifetime creates cumulative adversity, increasing their risk of depressive symptoms. We hypothesize that those with disadvantaged sociodemographic characteristics like household poverty, chronic health conditions, and negative lifecourse exposures will be more likely to report a high number of depressive symptoms in old age. Three sequential multivariable logistic regression models were estimated using Wave 3 (2012) of the Mexican Health and Aging Study (MHAS). The final sample included 5,610 respondents aged 50 and older, of which 34.3% reported depression measured by a modified 9-item CES-D scale. The sample was mostly female (63.3%) with a mean age of 69. Present-day conditions were measured by quality of the home, consumer durables, chronic health conditions, and health insurance. Early-life conditions were measured by the respondent’s mother’s education and exposure to poverty and illness during childhood. In 2012, older Mexican adults living in homes built with poor construction materials or homes that lack access to water and sanitation (OR=1.24) were more likely to experience high depressive symptoms. In addition, those living in homes without consumer durable goods (OR=1.23) were at increased risk. Older Mexican adults who experienced poverty (OR=1.16) or illness during childhood (OR=1.21) were more likely to report a high number of depressive symptoms in old age. In conclusion, we find evidence of a “long-arm” of childhood, whereas older Mexican adults’ exposure to poverty and illness in childhood increases their likelihood of poor mental health outcomes, regardless of their present-day conditions.

